# Suppression of top-down influence decreases both behavioral and V1 neuronal response sensitivity to stimulus orientations in cats

**DOI:** 10.3389/fnbeh.2023.1061980

**Published:** 2023-02-08

**Authors:** Zheng Ye, Jian Ding, Yanni Tu, Qiuyu Zhang, Shunshun Chen, Hao Yu, Qingyan Sun, Tianmiao Hua

**Affiliations:** ^1^College of Life sciences, Anhui Normal University, Wuhu, Anhui, China; ^2^School of Basic Medical, Wannan Medical College, Wuhu, Anhui, China

**Keywords:** primary visual cortex, top-down influence, area 7, orientation identification, orientation selectivity

## Abstract

How top-down influence affects behavioral detection of visual signals and neuronal response sensitivity in the primary visual cortex (V1) remains poorly understood. This study examined both behavioral performance in stimulus orientation identification and neuronal response sensitivity to stimulus orientations in the V1 of cat before and after top-down influence of area 7 (A7) was modulated by non-invasive transcranial direct current stimulation (tDCS). Our results showed that cathode (c) but not sham (s) tDCS in A7 significantly increased the behavioral threshold in identifying stimulus orientation difference, which effect recovered after the tDCS effect vanished. Consistently, c-tDCS but not s-tDCS in A7 significantly decreased the response selectivity bias of V1 neurons for stimulus orientations, which effect could recover after withdrawal of the tDCS effect. Further analysis showed that c-tDCS induced reduction of V1 neurons in response selectivity was not resulted from alterations of neuronal preferred orientation, nor of spontaneous activity. Instead, c-tDCS in A7 significantly lowered the visually-evoked response, especially the maximum response of V1 neurons, which caused a decrease in response selectivity and signal-to-noise ratio. By contrast, s-tDCS exerted no significant effect on the responses of V1 neurons. These results indicate that top-down influence of A7 may enhance behavioral identification of stimulus orientations by increasing neuronal visually-evoked response and response selectivity in the V1.

## Introduction

Although traditional views hold that visual information is encoded in a feed-forward fashion along hierarchical visual pathways (Hubel and Wiesel, 1968; Lee, 2002; Ro et al., 2003), a growing body of evidence indicate that top-down influence of high-level visual and even non-visual cortex on the low-level and primary visual cortex (V1) may play a critical role in visual perception and perceptual learning (Li et al., 2004; Lu and Dosher, 2004; Huang and Dobkins, 2005; Fenske et al., 2006; Eger et al., 2007; Rolls, 2008; Liu et al., 2010; Shibata et al., 2011; Gilbert and Li, 2013; Moldakarimov et al., 2014; Makino and Komiyama, 2015; Sharma et al., 2015; Kamiyama et al., 2016; Xiong et al., 2016; Pak et al., 2020). However, the mechanisms of top-down influence on neuronal response in the V1 remain in debate (Zhang et al., 2014; Kok et al., 2016; Klink et al., 2017; Nurminen et al., 2018; Cox et al., 2019; Keller et al., 2020; Ding et al., 2021; Federer et al., 2021). Moreover, how top-down influence concurrently affects behavioral detection of visual signals and V1 neuronal response is poorly understood (Zhang et al., 2014; Kirchberger et al., 2021; Ding et al., 2022).

A considerable number of studies have assessed the top-down influence on the neuronal response in the V1 or low-level visual cortex. Yet, the results reported by different authors are not consistent or even contradictory. Some authors suggest that top-down influence is primarily excitatory because most feedback connections to the V1 may use glutamate as neurotransmitters (Johnson and Burkhalter, 1994, 1997; van Loon et al., 2015; Ranson et al., 2019; Pan H. et al., 2021). Thus, top-down influence should enhance neuronal response and response sensitivity in the V1 (Wang et al., 2000, 2007, 2010, 2016; Galuske et al., 2002; Huang et al., 2004; Tong et al., 2011; Chen et al., 2014; Yang X. et al., 2016; Huh et al., 2018; Ding et al., 2021; Foster et al., 2021). Other authors show that top-down influence may activate inhibitory neuronal circuitry in the V1 and may exert suppressive effect on V1 neurons (Roland et al., 2006; Chalk et al., 2010; Nassi et al., 2013; Hishida et al., 2019; Maniglia et al., 2019). Still others even report a bidirectional top-down effect of both facilitation and inhibition on V1 neurons (Nurminen et al., 2018; Cox et al., 2019). It is unclear why results concerning top-down effects varied among different reports. Two factors may contribute. First, top-down influence may vary with cortical regions or species depending on characteristic network connections between the high-level and V1 (Payne, 1993; Johnson and Burkhalter, 1997; Budd, 1998; Connolly et al., 2012; Pan H. et al., 2021). Second, top-down effects may differ and change when top-down influence is modulated by different techniques, such as cortical lesion (Yang X. et al., 2016), cooling (Wang et al., 2000, 2007, 2010; Nassi et al., 2013), pharmacology administration (Tong et al., 2011; Jansen-Amorim et al., 2012; Chen et al., 2014; Yang X. et al., 2016; Hishida et al., 2019), optogenetic stimulation (Zhang et al., 2014; Nurminen et al., 2018) and attention modulation (Sharma et al., 2015; Cox et al., 2019; Foster et al., 2021), which could likely cause variations of top-down effect in time course, strength, and reversibility. Therefore, more studies using non-invasive and reversible tools are needed to extensively examine the top-down influence in different cortical regions and animal species.

On the other hand, most previous studies fail to assess top-down influence on both behavioral detection ability and neuronal response in the V1 simultaneously. Some studies have examined top-down influence on the response of V1 neurons but not on behavioral performance in visual signal detection (Hupé et al., 1998; Wang et al., 2000, 2007, 2010; Huang et al., 2004; Bardy et al., 2009; Thiele et al., 2009; Chalk et al., 2010; Tong et al., 2011; Al-Aidroos et al., 2012; Nassi et al., 2013; Chen et al., 2014; Kok et al., 2016; Yang X. et al., 2016; Huh et al., 2018; Cox et al., 2019; Federer et al., 2021). Others have measured top-down effect on behavioral performance but not on neuronal response and response sensitivity in the V1 (Ro et al., 2003; Huang and Dobkins, 2005; Rolls, 2008; Lu et al., 2011; Duque et al., 2013; Volberg et al., 2013; Cutrone et al., 2014). Therefore, the relationship between top-down influence on behavioral performance and on V1 neuronal activity is not fully understood (Zhang et al., 2014; Kirchberger et al., 2021), which is, however, critical to settle the debate about relative contribution of V1 and high-level cortical areas to visual perception (Koivisto et al., 2010; Ffytche and Zeki, 2011; Silvanto, 2014; Seidemann and Geisler, 2018).

Numerous studies have shown that transcranial direct current stimulation (tDCS) is a reliable non-invasive tool that can reversibly modulate neuronal excitability in the stimulated local brain region, with anode (a)- and cathode (c)-tDCS, respectively, enhancing and suppressing neuronal activity for a long-lasting (60–90 min) effect (Nitsche and Paulus, 2001; Schweid et al., 2008; Stagg et al., 2009; Monte-Silva et al., 2010; Bachtiar et al., 2015). Our recent investigations also demonstrate that c- and a-tDCS with the current intensity of 1 mA and a period of 15 min, respectively, decreases and enhances neuronal excitability with the effect confined in the stimulated cortical area and lasted for 60–70 min (Zhao et al., 2020; Ding et al., 2021, 2022; Pan D. et al., 2021). The current study will use tDCS tool to modulate top-down influence and observe concurrent change of both behavioral performance in orientation identification and the response selectivity of V1 neurons for stimulus orientations.

The cortical area 7 (A7) of cat is located on the middle suprasylvian gyrus and the adjacent lateral bank of the lateral sulcus, which receives a wide range of feedforward neural connectivity from area 19, 20a, 20b, 21a, 21b, AMLS, ALLS, and PLLS (Olson and Lawler, 1987; Connolly et al., 2012), and thus is defined as a higher-order extrastriate visual cortical area (Hicks et al., 1988). Further, neuronal tracing studies show that A7 had direct feedback connections to area 17 of the V1 (area 17) cortex, and the feedback neurons are primarily pyramidal cells that are distributed in discontinuous and sequential patches in layers 1, 2, and 3 or layer 5 of A7 (Han et al., 2008; Yang X. et al., 2016). Further, a study using fMRI indicates that inactivation of A7 with local injection of GABA or liquid nitrogen lesion results in a spatial frequency-dependent reduction in response amplitude of orientation maps in V1 (Yang X. et al., 2016). These evidences demonstrate that A7 is a high-level visual cortical area that may have direct excitatory top-down influence on V1 (Ding et al., 2021).

This study concurrently examines the behavioral threshold in stimulus orientation identification and the response selectivity of V1 neurons before and after the top-down influence is suppressed by tDCS in A7. We attempt to assess if the top-down influence exhibits a consistent effect on behavioral detection of visual signals and neuronal response in the V1.

## Materials and methods

### Subjects

Two adult male cats (age 3–5 years, body weight of 3.4–3.9 kg) were used in the current study. All cats were purchased from Nanjing Qing-Long-Shan Animal Breeding Farm (Jiangning District of Nanjing, Certificate No. SX1207), and all of them were disease-free, healthy cats with no optical or retinal abnormality. All animals were reared in rooms separated by transparent glass walls. Each room had comfortably organized living, feeding and playing areas with the room temperature maintained at 25°C. All experiments in this study were performed strictly in accordance with the National Institutes of Health Guide for the Care and Use of Laboratory Animals, and conformed to the principles and regulations as described in the ARRIVE guidelines (Animal Research: Reporting of *In Vivo* Experiments). All experiments and animal treatments were approved by the Ethics Committee of Anhui Normal University (approval NO. NS2017001).

### Conditioning training

The behavior training apparatus and procedures were similar to those described previously (Vandenbussche and Orban, 1983; De Weerd et al., 1990; Hua et al., 2010; Meng et al., 2013; Ding et al., 2022). Briefly, cats were trained to identify the orientation of a vertically- or horizontally-oriented grating on the display by touching the left (for vertical gratings) or right (for horizontal gratings) nose key to get fish mush reward (Supplementary video). The vertical and horizontal gratings had a fixed contrast of 100% but a varied spatial frequency (0.4 or 0.6 cycle/deg) during training so as to avoid that cats might detect gratings based on stimulus position cues rather than orientation. The vertical and horizontal gratings were randomly presented in each trial with an inter-trial interval of 2.5 s. The duration of each grating presentation was 2.35 s, including a denied period (RDP) of 0.35 s during which nose key touch was not rewarded. Prior to each stimulus presentation, a flashing dot (0.2° × 0.2°) appeared at the center of the CRT as a cue for the cat to fixate. Each cat performed 600–720 trials per day, arranged in 10–12 training blocks. Each block contained 60 trials, with a 2-min resting period between blocks. The conditioning training ended after ≥90% correct performance was attained.

### Administration of transcranial direct current stimulation

After success of conditioning training, a 3D printed plastic rectangle-shaped chamber (8 × 6 × 10 mm) was implanted on the skull over A7 (Horsley–Clarke coordinates: A0–A8/L6–L12) (Han et al., 2008; Yang X. et al., 2016; Ding et al., 2021, 2022) of the left hemisphere using dental cement. The surgery was performed after the animals were anesthetized and maintained in normal physiological state. The cat was first anesthetized with ketamine HCl (40 mg/kg, im) and xylazine (2 mg/kg, im). Non-invasive intubation of tracheal and intravenous cannula was performed under sterile preparation. After the cat was fixed in a stereotaxic apparatus, glucose (5%)-saline (0.9%) solution containing a mixture of urethane (20 mg/kg body weight) was infused intravenously to maintain necessary anesthesia. Artificial respiration was carried out, maintaining the expired pCO2 at approximately 3.8%. Heart rate (approximately 180–220 pulses/min) and electrocardiogram were monitored throughout the surgery process to assess the level of anesthesia and ensure that the animals were not responding to pain. The body temperature (38°C) was maintained using a heating blanket. At the end of the surgery, all incisions around the trauma were closed and sutured. Intravenous infusion was terminated first, and artificial ventilation was disconnected once the animal recovered spontaneous breathing. The animals received full care in the subsequent two weeks. Antibiotic (*penicillin*, 800,000 units per day) was administered (im) for about 2–3 days as needed. Behavioral measurement of stimulus orientation identification started after the cats recovered completely from the trauma.

The cathode (c)- and sham (s)-tDCS in A7 was applied through the implanted tDCS chamber using the same procedure as described previously (Zhao et al., 2020; Pan D. et al., 2021; Ding et al., 2022). Briefly, tDCS was administered with an HD-tDCS stimulator (Soterix Medical, New York, USA). A metal pin-type electrode (cathode) was placed in the tDCS chamber filled with 0.9% saline for conductance. The reference electrode (saline-soaked rubber electrode, 3 × 3 cm) was placed on the dorsal central neck skin after the hair over the intended site was clipped and cleaned with alcohol swabs. The output current intensity was maintained at 1 mA. At the onset and offset of stimulation, current was slowly ramped up and down over about 15 s to avoid sudden current change (Schweid et al., 2008; Wilson et al., 2018; Zhao et al., 2020; Pan D. et al., 2021). For control s-tDCS, the tDCS current was ramped down to zero after ramping up at the onset of stimulation, but ramped up and ramped down again in the end of sham stimulation. During daily behavioral measurement, the application of c- and s-tDCS in A7 was performed in a pseudorandom order with an interval of at least 90 min between different tDCS conditions so as to avoid tDCS effect interactions (Nitsche and Paulus, 2001; Stagg et al., 2009, 2011; Monte-Silva et al., 2010; Bachtiar et al., 2015; Zhao et al., 2020; Ding et al., 2022). The duration of each tDCS session was 15 min.

### Measurement of behavioral identification of stimulus orientations

**FIGURE 1:**
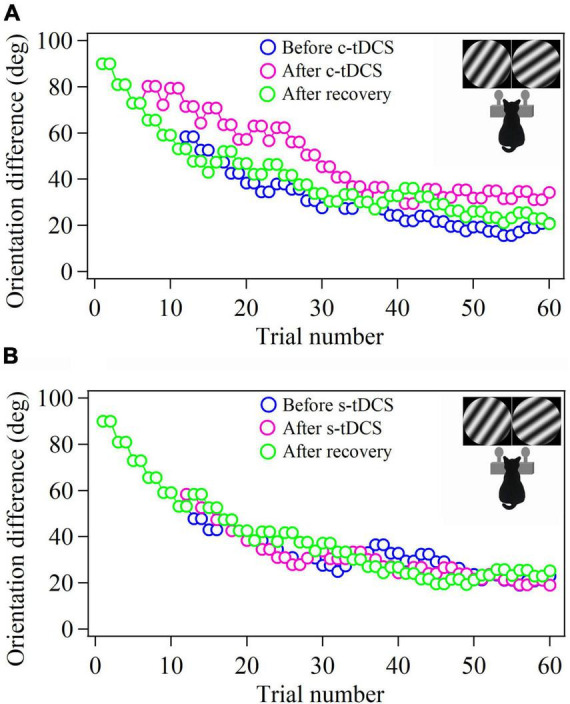
Samples showing the 2-correct down/1-error up staircase procedure that measures the behavioral threshold of cat in identifying stimulus orientation difference before and after tDCS in A7 as well as after withdrawal (or recovery) of tDCS effect. The c- and s-tDCS represent cathode **(A)** and sham tDCS **(B)**, respectively. Each procedure contains 60 trials. The schematic pictures of the cat in insert show the cat identifies the gratings with ±θ° approaching clockwise and counterclockwise to the 45° axis by touching, respectively, the left (clockwise) and right (counterclockwise) nose key to get food reward.

To evaluate top-down influence of A7 on the cat’s behavioral performance in identifying stimulus orientations, we measured the threshold of orientation difference (TO) that cats could identify grating stimuli with ±θ° approaching 45° or 135° axis from the vertical and horizontal orientation using the 2/1 staircase (d′ = 1.089) method before and after tDCS in A7 as well as after recovery of tDCS effect (Figure 1). Daily measurements for c- and s-tDCS condition were arranged in a pseudorandom order with an interval of at least 90 min. Measurement at each condition contained 60 trials, which was completed within 5 min before tDCS, after tDCS and after recovery of tDCS effect (at least 90 min after the end of tDCS application) (Zhao et al., 2020; Ding et al., 2022), respectively. Statistical difference in the average TO across eight repeated daily measurements before and after c- or s-tDCS was determined using ANOVA and *Post hoc* test.

At the end of each daily measurement, the cats were provided with supplemental ordinary food according to the food requirement during conditioning training.

### Examination of the response and response selectivity of V1 neurons

#### Recording preparation

The recording procedure was similar to that described previously (Hua et al., 2010; Meng et al., 2013; Yang J. et al., 2016; Zhao et al., 2020; Ding et al., 2021; Pan D. et al., 2021). After being anesthetized with ketamine HCl (40 mg/kg, im) and xylazine (2 mg/kg, im), the cat was intubated with tracheal and intravenous cannula under sterile preparation. Then the cat was fixed in a stereotaxic apparatus, and maintained in anesthesia and paralysis state through intravenous infusion of glucose (5%)-saline (0.9%) solution containing a mixture of urethane (20 mg/kg body weight) and gallamine triethiodide (10 mg/kg body weight). Artificial respiration was performed, and the expired pCO2 was kept at approximately 3.8%. The animal’s heart rate (180–220 beats/min), electrocardiogram, and blood oxygen level (>95%) were recorded continuously during the experiment to monitor the anesthesia level and physiological state. The body temperature (38°C) was maintained using a heating blanket. Pupils were maximally dilated with atropine (0.5%), and artificial tear was applied to protect the cornea.

The V1 area (Horsley–Clarke coordinates: P0-8/L0-4) of the left hemisphere was exposed by performing a craniotomy on the skull under a microscope. The dura over V1 was cut and removed. The exposed V1 was covered with a 4% agar saline solution. Multi-unit recording in the V1 was done using a glass-coated tungsten microelectrode (with an impedance of 3–5 MΩ), which was driven by a hydraulic micromanipulator (Narishige, Tokyo, Japan). Multi-units were randomly sampled from all cortical layers in the medial bank of the lateral gyrus with the electrode penetrations within a vertical depth of 2000 μm from the pial surface (Supplementary Figure 1). The distance between penetrations and recording locations of different multi-units were kept at least 200 μm apart. Action potentials of the recorded multi-units were amplified (×2,000) by a microelectrode amplifier (Dagan 2400A, Minneapolis, MN, USA) and then fed into a window discriminator with an audio monitor. The original voltage traces were digitized by an acquisition board (National Instruments, Austin, TX, USA) controlled by IGOR software (WaveMetrics, Portland, OR, USA) and then saved for on- or off-line analysis.

#### Visual stimuli and recording procedure

Visual stimuli were drifting sinusoidal grating generated in MATLAB with the aid of high-level Psychophysics Toolbox (Brainard, 1997). Once the visually-evoked response of a multi-unit was detected, the receptive field center of the multi-unit was preliminarily determined using bars of light emitted from a hand pantoscope and then precisely mapped by presenting repeatedly a series of computer-generated flashing bars of light on a movable CRT monitor (resolution 1024 × 768, refresh rate 75 Hz) positioned 57 cm from the cat’s eyes. We selected optimal stimulus size, temporal, and spatial frequency for each multi-unit. Each stimulus was presented to the dominant eye. Multi-unit response selectivity bias for stimulus orientations before and after tDCS in A7 as well as after recovery of tDCS effect (90 min after the end of tDCS) was evaluated by presenting a series of grating stimuli with different orientations (0–180° scale with an increment of 15°) moving in two directions. Each stimulus was randomly presented and repeated 3–4 times. Before each stimulus presentation, the spontaneous activity was obtained while mean luminance was shown on the display for 1 s. The contrast for each stimulus was set at 100%. The mean luminance of the display was 19 cd/m^2^, and the environmental luminance on the cornea was 0.1 lx. The application of c- and s-tDCS in A7 were interleaved for different multi-units, with an interval of at least 90 min between different tDCS sessions. The tDCS procedure, intensity and duration were the same as described above in the Methods of “Administration of transcranial direct current stimulation.”

At the end of electrophysiological recording, the cat was deeply anesthetized with ketamine HCl (80 mg/kg, im) and xylazine (4 mg/kg), and then transcardially perfused with 0.9% saline followed by 2% paraformaldehyde in 0.1 M phosphate buffered saline (PBS) according to methods described previously (Yang J. et al., 2016; Pan H. et al., 2021). The brain tissue containing visual cortical areas was removed and post-fixed in 2% paraformaldehyde for subsequent histological examination. Briefly, the cerebral cortex containing visual cortical area 17, 18, 19, 21a, PMLS, and 7 was dissected and cryoprotected by sequential incubation in 10% (2 h), 20% (2 h), and 30% (overnight) sucrose until tissue sinking. Then, the brain tissue was embedded in OCT compound (Tissue-Tek, 4583, Sakura Finetek Inc., California, USA), and coronal sections were cut at a thickness of 40 μM using a Leica cryostat (Leica Biosystems Inc., Buffalo Grove, IL, USA). Serial frozen sections were collected in order, placed in wells filled with cryoprotectant solution (ethylene glycol-based; 30% ethylene glycol, 30% sucrose, 1% PVP-40, in 0.1 M Phosphate buffer pH 7.4) and temporarily stored at −20°C for subsequent immunoreactive labeling of cortical neurons. The free-floating sections were first incubated overnight at 4°C with rabbit anti-NeuN (1:1000, ab177487, Abcam, Shanghai, China). After several washes in PBS, sections were incubated with the secondary antibody (goat anti-Rabbit IgG H&L, Alexa Fluor 488, 1:1000, ab150077; Abcam) diluted in QuickBlock Secondary Antibody Dilution Buffer (P0265; Beyotime) for 2 h at room temperature. After further washes in PBS, sections were mounted on clean glass slides with glycerol and sealed with nail polish. Images were taken under Leica inverted fluorescent microscope (DMi8 automated, Leica, Germany) using 10× objective.

#### Data collection and analysis

All data analysis was performed based on the multi-unit response to visual stimuli. The multi-unit response to a grating stimulus was defined as the mean firing rate (spontaneous response subtracted) corresponding to the time of stimulus presentation, which was used to plot the tuning curves of the multi-unit response to stimulus orientations, temporal, and spatial frequencies.

The response of each multi-unit to different stimulus orientations (with two motion directions at each orientation) was fitted with a double Gaussian function for providing a qualitative description of the tuning curve but not for analysis of orientation selective index (Pattadkal et al., 2018):


(1) $$R\,\left(\theta \right)=A_{0}+A_{1}\times e^{\frac{-{\left(\theta -\theta_{\text{pref}}\right)}^{2}}{2\,\sigma^{2}}}+A_{2}\times e^{\frac{-{\left(\theta -\theta_{\text{pref}}-\pi \right)}^{2}}{2\,\sigma^{2}}}$$

where R(θ) is the averaged response to a grating stimulus with motion direction θ; A_0_ is the mean offset across the four lowest points in the orientation tuning curve; A_1_ and A_2_ are the response amplitudes of the two Gaussians; θ_pref_ indicates the preferred orientation, and σ is the standard deviation of the Gaussian function.

**FIGURE 2:**
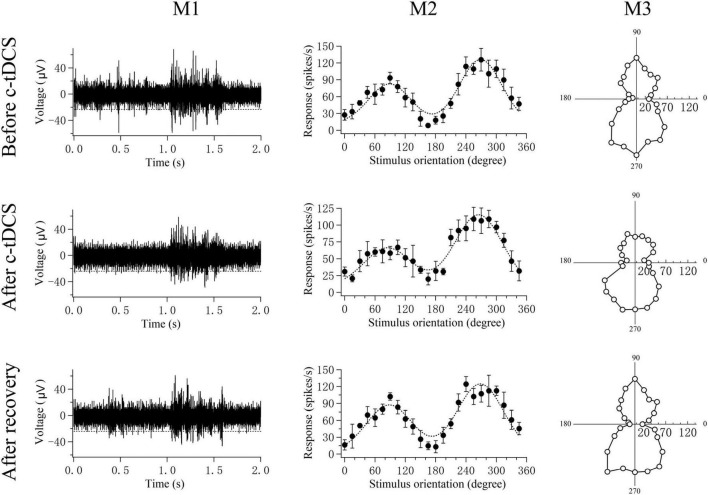
A sample showing the computation of a V1 multi-unit preferred orientation and response selectivity bias for different stimulus orientations before and after c-tDCS in A7 as well as after recovery of c-tDCS effect. M1 shows the voltage trace of the multi-unit response to the preferred-orientation stimulus (five trials). The spontaneous activity was acquired during the pre-stimulus time (0.0–1.0 s). Spikes above the broken lines were counted as action potentials; M2 shows the mean visually-evoked response of the multi-unit (filled circles with error bar of SD) to different stimulus orientations and motion directions as well as the orientation tuning curve (dotted curve) fitted with double Gaussian function (see section “Materials and methods”). M3 shows the plot of circle variance computing the orientation selectivity bias based on OSI function (see section “Materials and methods”). The responses of each multi-unit to the different stimulus orientations or directions were stored as a series of vectors. The vectors were added and divided by the sum of the absolute values of the vectors. The angle (0°–360°) of the resultant vector gives the preferred orientation or direction of the multi-unit. The length of the resultant vector provides a quantitative measure of the orientation or direction sensitivity bias of the multi-unit.

The preferred orientation and orientation selectivity bias for each multi-unit were computed using the orientation selectivity index (OSI) function based on the raw data of the multi-unit responses as described elsewhere (Schmolesky et al., 2000; Leventhal et al., 2003; Hua et al., 2006; Yang J. et al., 2016; Scholl et al., 2017; Pattadkal et al., 2018; Figure 2):


(2) $$O\,S\,I=\frac{\sqrt{{\left[\sum R\,\left(\theta \right)\,\sin\left(2\,\theta \right)\right]}^{2}+{\left[\sum R\,\left(\theta \right)\,\cos\left(2\,\theta \right)\right]}^{2}}}{\sum R\,\left(\theta \right)}$$

where θ is the motion direction of the grating stimuli, and *R*(θ) is the mean response at motion direction θ. Briefly, the responses of each multi-unit to the different stimulus orientations or directions were stored as a series of vectors. The vectors were added and divided by the sum of the absolute values of the vectors. The angle of the resultant vector gave the preferred orientation or direction of the multi-unit. The length of the resultant vector, termed the orientation or direction bias, provided a quantitative measure of the orientation or direction sensitivity of the multi-unit.

The signal-to-noise ratio (STN) of a multi-unit was defined as the ratio between the maximum visually evoked response and the spontaneous activity. All data were expressed as mean ±SD. Statistical difference before and after c- or s-tDCS was performed using repeated two-way ANOVA and *Post hoc* tests with least significant difference (LSD).

## Results

### Top-down influence on behavioral identification of stimulus orientations

We first evaluated the effect of top-down suppression on the behavioral performance in stimulus orientation detection by measuring the threshold of orientation difference (TO) that cats could identify stimulus orientations with ±θ° from 45° or 135° axis before and after c- or s-tDCS in A7 using 2/1 staircase method (Figure 1).

**FIGURE 3:**
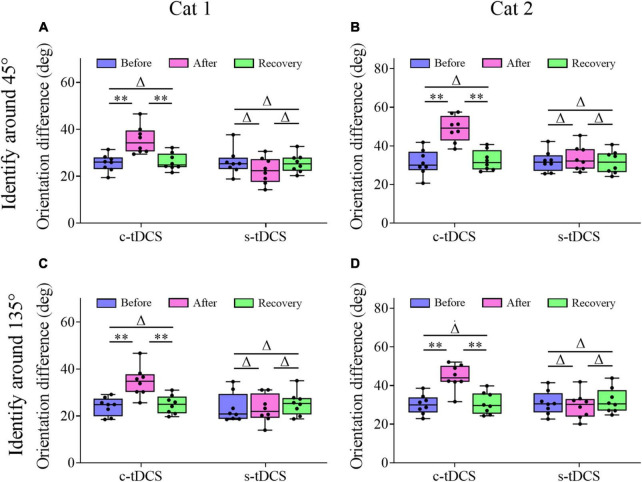
Whisker diagrams showing the behavioral threshold of orientation difference that cat1 **(A,C)** and cat2 **(B,D)** can identify grating stimuli around the 45° **(A,B)** and 135° **(C,D)** axis before and after c- or s-tDCS in A7 as well as after withdrawal (recovery) of tDCS effect. ^Δ^*p* > 0.05, **p < 0.0001. The box plots show the median (middle line within box), 25th–75th percentiles (box), minimum and maximum values (whiskers). The solid dots represent individual data.

Two-way ANOVA showed that the mean TO value measured around the 45° axis before c-tDCS, immediately after c-tDCS and after withdrawal (recovery) of tDCS effect (90 min after the end of tDCS) had significant difference [F(2,48) = 31.623, *p* < 0.0001], and the c-tDCS effect had no significant interaction with cat [F(2,48) = 2.467, *p* = 0.097] (Figures 3A, B). Further *Post hoc* test (LSD) indicated that the mean TO around the 45° axis measured immediately after c-tDCS was significantly larger than that before c-tDCS in A7 (*p* < 0.0001), whereas the mean TO measured after recovery of tDCS effect exhibited no significant difference from that measured before c-tDCS (*p* = 0.692) but was significantly lower than that measured immediately after c-tDCS in A7 (*p* < 0.0001). By contrast, the mean TO around the 45° axis measured before s-tDCS, after s-tDCS and after recovery of tDCS effect showed no significant variation [F(2,48) = 0.126, *p* = 0.882], and there was no interaction between tDCS effect and subject [F(2,48) = 1.161, *p* = 0.323] (Figures 3A, B). *Post hoc* test also indicated that the mean TO around the 45° axis measured immediately after s-tDCS had no significant variation from that before s-tDCS in A7 (*p* = 0.619), and the mean TO measured after recovery of tDCS effect displayed no difference from that measured before s-tDCS (*p* = 0.82) and immediately after s-tDCS (*p* = 0.787) either.

Similarly, Two-way ANOVA analysis indicated that the mean TO value measured around the 135° axis before and after c-tDCS in A7 as well as after recovery of tDCS effect displayed significant difference [F(2,48) = 27.211, *p* < 0.0001], and the c-tDCS effect exhibited no interaction with subject [F(2,48) = 0.65, *p* = 0.527] (Figures 3C, D). Further *Post hoc* test showed that the mean TO value measured around the 135° axis increased significantly after c-tDCS relative to before c-tDCS (*p* < 0.0001), whereas the mean TO value measured after withdrawal of c-tDCS effect had no significant difference from that before c-tDCS in A7 (*p* = 0.703) but was significantly smaller than that measured immediately after c-tDCS (*p* < 0.0001). By contrast, the mean TO measured around the 135° axis before and after s-tDCS in A7 as well as after recovery time of tDCS effect had no significant difference [F(2,48) = 0.616, *p* = 0.545], and there was no interaction between tDCS effect and subject [F(2,48) = 0.033, *p* = 0.967] (Figures 3C, D). *Post hoc* test showed that the mean TO around the 135° axis measured after s-tDCS was not different from that before s-tDCS (*p* = 0.684), and the mean TO measured after withdrawal of tDCS effect had no variation from that measured before s-tDCS (*p* = 0.495) and immediately after s-tDCS (*p* = 0.278).

The comparisons above showed that c-tDCS in A7 displayed a comparable suppressive effect on behavioral detection of stimulus orientation difference around 45°- and 135°-axis, which indicated that top-down influence of A7 on the behavioral performance in stimulus orientation identification could be generalized to different orientation axes.

### Top-down influence on the response selectivity of V1 neurons

After behavioral measurement of orientation identification, we recorded the response of multi-units in V1 to stimuli with different orientations before and after tDCS in A7 as well as after withdrawal of tDCS effect (90 min after the end of tDCS), so as to evaluate if suppression of top-down influence exerted a consistent effect on the response selectivity of V1 neurons with the effect on behavioral detection. A total of 47 multi-units (25 multi-units for s-tDCS and 22 multi-units for c-tDCS) in cat1 and 50 multi-units (27 multi-units for s-tDCS and 23 multi-units for c-tDCS) in cat2 were recorded at 8–10 randomly selected electrode penetrations in the V1 (Supplementary Figures 1A, B). Histological examination confirmed that all penetrations were within the gray matter of V1 (Supplementary Figure 1C).

**FIGURE 4:**
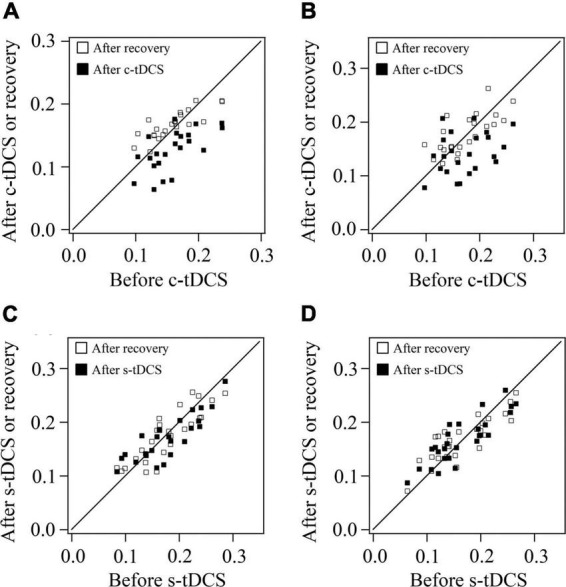
Scatter plots showing the orientation selectivity bias (OB) of multi-units recorded in V1 cortex after versus before c-tDCS **(A,B)** or s-tDCS **(C,D)** as well as after recovery of tDCS effect versus before tDCS in A7 of cat1 **(A,C)** and cat2 **(B,D)**, respectively.

As shown in the scatter plot for orientation selectivity bias (OB) of V1 multi-units in both cats, the OB value of most multi-units from both cats reduced after c-tDCS relative to before c-tDCS in A7, and nearly recovered to the original value after withdrawal of tDCS effect (Figures 4A, B). Two-way ANOVA analysis indicated that there was a significant difference among the mean OB values measured before c-tDCS, after c-tDCS and after withdrawal of tDCS effect [F(2,135) = 14.467, *p* < 0.0001]; the effect had no significant interaction with cat [F(2,135) = 0.056, *p* = 0.945]. Further *Post hoc* test (LSD) showed that the mean OB of V1 multi-units was significantly reduced after c-tDCS relative to before c-tDCS in A7 (*p* < 0.0001) whereas the mean OB of V1 multi-units after withdrawal of c-tDCS effect had no significant variation from that before c-tDCS (*p* = 0.376) but was significantly higher than that after c-tDCS in A7 (*p* < 0.0001). By contrast, the OB value of most multi-units in both cats after s-tDCS or after withdrawal of tDCS effect was identical or close to that before s-tDCS in A7 (Figures 4C, D). Two-way ANOVA analysis showed that the mean OB value of V1 multi-units measured before and after s-tDCS in A7 as well as after recovery time of tDCS effect exhibited no significant variation [F(2,156) = 0.048, *p* = 0.953], and there was no significant interaction between tDCS effect and cat [F(2,156) = 0.124, *p* = 0.884]. These comparisons indicated that suppression of top-down influence of A7 decreased the response selectivity of V1 multi-units for stimulus orientations, which could recover after withdrawal of top-down influence inhibition. This effect was consistent with the top-down influence on behavioral performance in stimulus orientation identification.

A lowered response selectivity of V1 multi-units for stimulus orientations could be caused by changes in their preferred orientation or visually evoked response or spontaneous activity. To examine these possibilities, we further compared the preferred orientation, visually-evoked response and spontaneous activity of multi-units recorded in the V1 before and after tDCS in A7.

**FIGURE 5:**
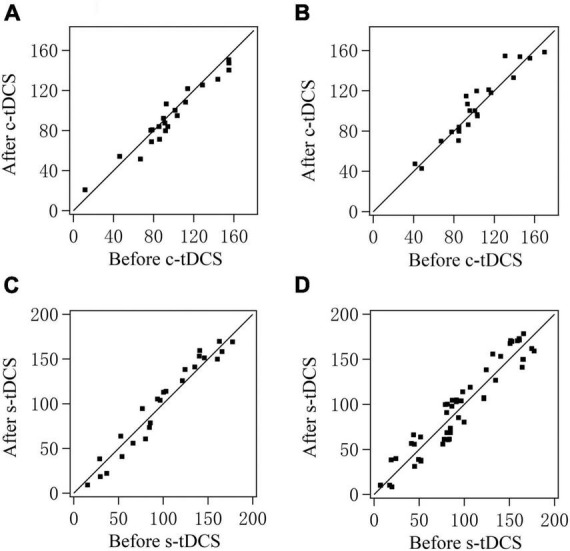
Scatter plots showing the preferred orientation of multi-units recorded in V1 cortex after versus before c-tDCS **(A,B)** or s-tDCS **(C,D)** in A7 of cat1 **(A,C)** and cat2 **(B,D)**, respectively.

As shown in scatter plots, the preferred orientation (PO) of V1 multi-units in both cats after either c-tDCS (Figures 5A, B) or s-tDCS (Figures 5C, D) was identical or quite close to that before tDCS. ANOVA analysis showed that the mean PO of V1 multi-units measured after c-tDCS was not significantly different from that before c-tDCS in A7 [F(1,90) = 0.018, *p* = 0.895], and there was no interaction between tDCS effect and subject [F(1,90) = 0.126, *p* = 0.723]. Again, the mean PO of V1 multi-units measured after s-tDCS had no significant difference from that before s-tDCS in A7 [F(1,104) = 0.014, *p* = 0.905], and the effect exhibited no interaction with cat [F(1,104) = 0.0001, *p* = 0.99]. These results indicated that suppression of top-down influence from A7 had no significant impact on the PO of V1 multi-units.

**FIGURE 6:**
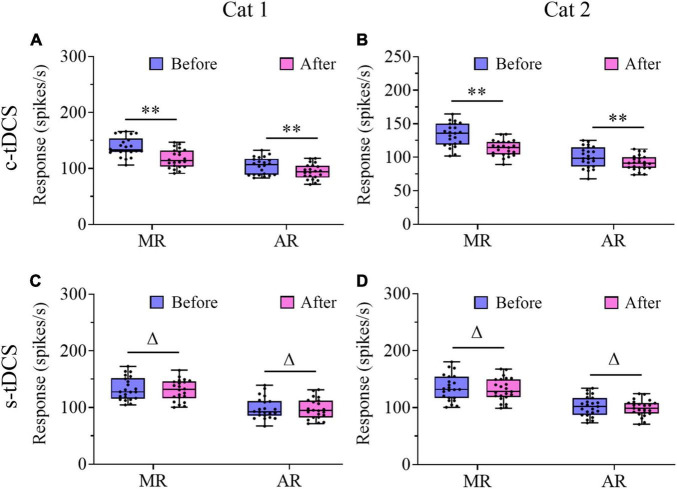
Whisker diagrams showing the median (middle lines within box), 25th–75th percentiles (box), minimum and maximum values (whiskers) of the maximum response (MR) and averaged response across all stimulus orientations (AR) of V1 multi-units before and after c-tDCS **(A,B)** or s-tDCS **(C,D)** in A7 of cat1 **(A,C)** and cat2 **(B,D)**, respectively. The solid dots represent individual data. ^Δ^*p* > 0.05, **p < 0.01.

We further compared the visually-evoked response and spontaneous activity of V1 multi-units before and after tDCS in A7. Two-way ANOVA analysis showed that the mean maximum visually-evoked response (MR) of V1 multi-units after c-tDCS in A7 was significantly lowered when compared with that before c-tDCS [F(1,90) = 37.894, *p* < 0.0001], and the effect was independent of subject [F(1,90) = 0.021, *p* = 0.886] (Figures 6A, B). By contrast, the mean MR of V1 multi-units after s-tDCS in A7 had no significant difference from that before s-tDCS [F(1,104) = 0.19, *p* = 0.664], and the effect was independent of cat [F(1,104) = 0.131, *p* = 0.718] (Figures 6C, D). Similarly, Two-way ANOVA indicated that the average visually-evoked response (AR) across all stimulus orientations of V1 multi-units after c-tDCS in A7 was significantly lower than before c-tDCS [F(1,90) = 10.081, *p* = 0.002], which effect was independent of subject [F(1,90) = 0.074, *p* = 0.787] (Figures 6A, B). By contrast, the mean AR exhibited no significant variation after s-tDCS relative to before s-tDCS in A7 [F(1,104) = 0.165, *p* = 0.685] with no significant interaction with cat [F(1,104) = 0.007, *p* = 0.936] (Figures 6C, D). Relative to before c-tDCS, the mean MR decreased by 15.4% and 14.9% in cat1 and cat2, respectively, whereas the mean AR decreased by 9.7% and 8.6% in cat1 and cat2, respectively.

**FIGURE 7:**
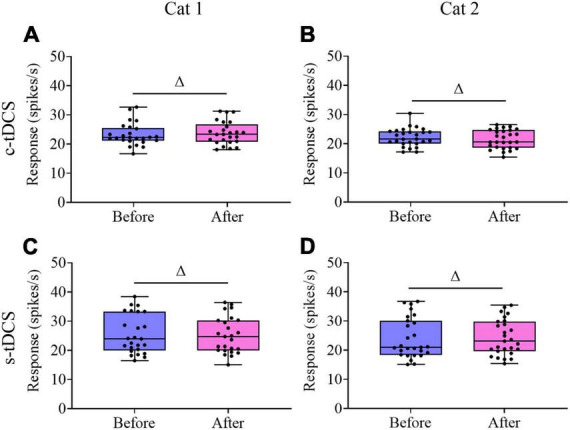
Whisker diagrams showing the median (middle lines within box), 25th–75th percentiles (box), minimum and maximum values (whiskers) of the spontaneous activity of V1 multi-units before and after c-tDCS **(A,B)** or s-tDCS **(C,D)** in A7 of cat1 **(A,C)** and cat2 **(B,D)**, respectively. The solid dots represent individual data. ^Δ^*p* > 0.05.

**FIGURE 8:**
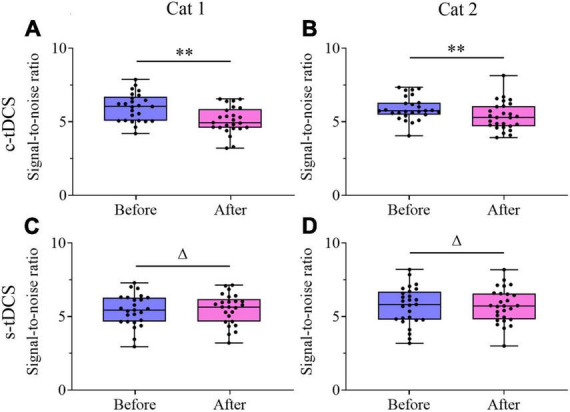
Whisker diagrams showing the median (middle lines within box), 25th–75th percentiles (box), minimum and maximum values (whiskers) of the signal-to-noise ratio of V1 multi-units before and after c-tDCS **(A,B)** or s-tDCS **(C,D)** in A7 of cat1 **(A,C)** and cat2 **(B,D)**, respectively. The solid dots represent individual data. ^Δ^*p* > 0.05, **p < 0.0001.

Unlike the visually-evoked response, Two-way ANOVA indicated that the mean spontaneous activity of V1 multi-units showed no significant change after either c-tDCS [F(1,90) = 0.001, *p* = 0.974] (Figures 7A, B) or s-tDCS [F(1,104) = 0.0001, *p* = 0.983] (Figures 7C, D) relative to before tDCS in A7, and the effect had no interaction with subject [c-tDCS: F(1,90) = 0.537, *p* = 0.466; s-tDCS: F(1,104) = 0.076, *p* = 0.784]. As a result, the mean signal-to-noise ratio (STN) of V1 multi-units was significantly lowered after c-tDCS relative to before c-tDCS [c-tDCS effect: F(1,90) = 21.531, *p* < 0.0001; Interaction of c-tDCS and cat: F(1,90) = 0.676, *p* = 0.413] (Figures 8A, B), whereas the mean STN of V1 multi-units after s-tDCS had no significant difference from that before s-tDCS [s-tDCS effect: F(1,104) = 0.032, *p* = 0.859; Interaction of s-tDCS and cat: F(1,104) = 0.019, *p* = 0.891] (Figures 8C, D).

All comparisons outlined above indicated that suppression of top-down influence with c-tDCS in A7 significantly decreased the visually-evoked response especially the maximum response of V1 neurons but not spontaneous activity, which caused a weakness in response selectivity for different stimulus orientations and a reduction in signal-to-noise.

## Discussion

How top-down influence from high-level cortical areas affects behavioral detection of visual signals and neuronal response in the V1 is poorly understood. This study examined both behavioral and V1-neuronal response sensitivity of cats to stimulus orientations before and after top-down influence of A7 was suppressed with c-tDCS. The results showed that suppression of top-down influence of A7 significantly increased the behavioral threshold in identifying stimulus orientation difference, which effect could recover after withdrawal of tDCS effect. Consistently, suppression of top-down influence of A7 lowered the visually-evoked response especially the maximum response of V1 neurons and thus decreased the response selectivity to stimulus orientations, which effect could also recover after tDCS effect vanished. These results indicate that top-down suppression-induced reduction of V1-neuronal response and response selectivity may underline the weakened behavioral performance in orientation identification following inhibition of top-down influence.

### Top-down influence on the response and response selectivity of V1 neurons

Examining top-down effects of higher-level cortex on neuronal response and response selectivity in the low-level and V1 is critical for understanding how top-down influence mediates visual perception. Although it has been widely reported that top-down influence affects the response of V1 neurons in several mammalian species, the results from different studies are diverse. Some studies show that top-down influence facilitates neuronal response in the V1 (Wang et al., 2000, 2007, 2010, 2016; Galuske et al., 2002; Tong et al., 2011; Keller et al., 2020; Ding et al., 2021; Foster et al., 2021) or differentially increases neuronal response depending on their preferred spatial frequencies (Huang et al., 2004; Yang X. et al., 2016). Other studies report suppressive top-down influence on V1 neuronal response (Nassi et al., 2013; Hishida et al., 2019; Maniglia et al., 2019), and still others have observed multiple effects of both facilitation and suppression (Gazzaley et al., 2005; Cox et al., 2019). The observed top-down effects on the response selectivity of V1 neurons are also inconsistent. Some authors have found that top-down influence primarily affects orientation selectivity (Wang et al., 2000, 2007; Huang et al., 2004; Tong et al., 2011; Yang X. et al., 2016; Hishida et al., 2019). Others suggest top-down effects on motion direction selectivity (Galuske et al., 2002; Huang et al., 2017) but not on orientation selectivity (Chen et al., 2014). Still others report top-down influence on both orientation and motion direction selectivity (Jansen-Amorim et al., 2012).

Reasons underlying the inconsistency of observed top-down effects are unclear. An important factor could be that top-down influence is modulated by different methods in different studies. Although cortical cooling (Wang et al., 2000, 2007, 2010; Bardy et al., 2009; Nassi et al., 2013), pharmacological driving (Huang et al., 2004; Tong et al., 2011; Jansen-Amorim et al., 2012; Chen et al., 2014; Yang X. et al., 2016; Hishida et al., 2019), TMS/tDCS control (Maniglia et al., 2019; Ding et al., 2021), and optogenetic manipulation (Zhang et al., 2014; Huh et al., 2018; Nurminen et al., 2018; Kirchberger et al., 2021) can all suppress neuronal activity in the target high-level cortex, the influence induced by different methods may vary in cortical range, time course, strength, and even reversibility of effect, which can cause variations in top-down influence. Attention control can non-invasively and reversibly modulate neuronal response in the low-level and V1 (Thiele et al., 2009; Chalk et al., 2010; Lee and Maunsell, 2010; Sharma et al., 2015; Wang et al., 2016; Alilović et al., 2019; Cox et al., 2019; Foster et al., 2021), but is susceptible to fluctuation depending on brain states (Gilbert and Sigman, 2007). The current study used a non-invasive tool of tDCS to reversibly modulate neuronal activity of A7 in cat (Ding et al., 2021, 2022), and examined the top-down influence on the response of V1 neurons immediately after tDCS and after withdrawal of tDCS effect. Our results showed that suppression of top-down influence with c-tDCS in A7 decreased the visually evoked response especially the maximum response but not spontaneous activity of V1 neurons, and thus reduced neuronal response selectivity for stimulus orientations, which effect could recover after tDCS effect vanished. Our results are consistent with some previous studies in cat (Wang et al., 2000, 2007, 2010; Tong et al., 2011) and mouse (Zhang et al., 2014; Huh et al., 2018), but different from some observations in primate (Jansen-Amorim et al., 2012; Nassi et al., 2013; Nurminen et al., 2018; Cox et al., 2019). Another factor leading to the current debate on top-down influence is likely related to the difference of observed cortical regions or species. It remains unclear whether and how the corticocortical feedback connections vary with cortical areas or even species. Although a few studies show that most feedback projections are glutamatergic fibers (Johnson and Burkhalter, 1994, 1997; van Loon et al., 2015; Pan H. et al., 2021), inhibitory feedback neurons may also exist (Budd, 1998; Pan H. et al., 2021). Moreover, excitatory top-down projections can activate inhibitory neural circuitry in the V1 either (Zhang et al., 2014; Kirchberger et al., 2021). Further studies are needed to clarify factors underlying these discrepancies and elucidate the mechanism of top-down influence on V1.

### Contribution of top-down influence to orientation identification

Although a considerable number of studies have observed top-down influence on visual perceptual detection or neuronal response in the V1, the correlation between top-down effects on behavior and neuronal response remains poorly understood. Some studies have assessed top-down influence especially attention-related influence on behavioral detection but not on neuronal response changes in the V1 (Lu and Dosher, 1998, 2004; Dosher and Lu, 2000; Huang and Dobkins, 2005; Carrasco, 2006; Fenske et al., 2006; Rolls, 2008; Cutrone et al., 2014; Wang et al., 2016). Other studies have examined top-down effect on the response of low-level and V1 neurons but not on behavioral identification of visual signals (Roland et al., 2006; Williford and Maunsell, 2006; Li et al., 2008; Thiele et al., 2009; Jansen-Amorim et al., 2012; Chen et al., 2014; Sharma et al., 2015; Yang X. et al., 2016; Huang et al., 2017; Huh et al., 2018; Nurminen et al., 2018; Cox et al., 2019; Hishida et al., 2019). Therefore, whether top-down influence exhibits a consistent effect on behavioral detection and the V1 neuronal activity is not clearly understood (Eger et al., 2007; Zhang et al., 2014; Sharma et al., 2015; Ding et al., 2022).

Orientation identification is critical in visual pattern and form perception, which depends on the fundamental response property of visual cortical neurons especially V1 neurons in stimulus orientation selectivity (Edden et al., 2009; Glickfeld et al., 2013; Li et al., 2019). Numerous studies have shown that top-down influence can significantly increase the response selectivity of V1 neurons for stimulus orientations (Wang et al., 2000, 2007; Galuske et al., 2002; Huang et al., 2004, 2017; Tong et al., 2011; Jansen-Amorim et al., 2012; Yang X. et al., 2016; Hishida et al., 2019). However, these studies fail to assess top-down influence on behavioral performance in orientation identification simultaneously due to the limitation of methods used for top-down effect modulation. The current study used non-invasive tDCS to modulate neuronal activity in the high-level visual cortex of A7, and examined top-down influence on both behavioral and V1 neuronal response sensitivity to stimulus orientations. Our results showed that suppression of top-down influence of A7 significantly increased the behavioral threshold in identifying stimulus orientation difference, and consistently decreased the response selectivity of V1 neurons for stimulus orientations. Furthermore, this top-down influence on both behavioral performance and neuronal response could recover after withdrawal of tDCS effect. Our results are consistent with the computational predictions of neural network models (Moldakarimov et al., 2014) and recent observations of top-down influence on visual contrast detection (Zhang et al., 2014; Ding et al., 2022). Taken together, these results indicate that top-down influence may enhance perceptual orientation identification by increasing the response selectivity of V1 neurons for stimulus orientations. In addition, our results provide a new evidence that highlights the fundamental role of V1 in behavioral detection of visual signals (Glickfeld et al., 2013; Seidemann and Geisler, 2018; Ding et al., 2022) and supports the reverse hierarchy theory that visual perception is based on information processing loops between V1 and higher-level cortical areas (Juan and Walsh, 2003; Tong, 2003; Juan et al., 2004; Ding et al., 2021).

### Limitations of the study

The current study showed that suppression of top-down influence from A7 had a consistent impact on behavioral identification of stimulus orientations and V1 neuronal response selectivity for stimulus orientations. There are several limitations needed to be clarified in the subsequent studies.

First, this study used cathode tDCS to suppress top-down influence of A7, and found that behavioral detection ability and V1 neuronal response selectivity were all decreased. Further studies should address if enhanced top-down influence with anode-tDCS (Stagg et al., 2009; Zhao et al., 2020) in A7 could increase both behavioral and neuronal performance, which will enable clinical application for improving visual function of human subjects (Spiegel et al., 2013; Ding et al., 2016; Zhang et al., 2020).

Second, the current study examined the response and response selectivity of V1 neurons in the anesthetized cat. It is unknown whether the tDCS-induced top-down effect on neuronal response property under awake state is consistent with that observed under anesthesia state. Subsequent studies should design new experiments to conduct behavioral measurement and electrophysiological recording simultaneously in awake animals (Zhang et al., 2014).

Finally, we have found significant top-down influence of A7 on the response and response selectivity of V1 neurons, the underlying neural substrate that mediates the top-down influence remains unclear. Although previous evidence has confirmed a direct corticocortical connection between A7 and V1 area (Han et al., 2008), future studies need to identify the types of feedback neurons as well as their target neurons in the V1 using combined techniques of neuronal tracing and double immunofluorescent labeling (Connolly et al., 2012; Pan H. et al., 2021) so as to elucidate neuronal mechanisms of top-down influence.

## Data availability statement

The raw data supporting the conclusions of this article will be made available by the authors, without undue reservation.

## Ethics statement

All experiments in this study were performed strictly in accordance with the National Institutes of Health Guide for the Care and Use of Laboratory Animals, and conformed to the principles and regulations as described in the ARRIVE guidelines (Animal Research: Reporting of *In Vivo* Experiments). All experiments and animal treatments were approved by the Ethics Committee of Anhui Normal University (approval NO. NS2017001).

## Author contributions

ZY, JD, and TH: study design. ZY, JD, and QS: cat behavioral training and psychophysical measurement of orientation identification threshold. ZY, JD, YT, QZ, SC, and HY: surgery and electrophysiological recording. YT and QZ: histological examination. All authors have made contributions to data interpretation, manuscript drafting and revising, and have approved the final version of the manuscript.

## Supplementary material

Supplementary Figure 1 Show the recording sites in the V1 cortex (area 17, A17) of cat1 **(A)** and cat2 **(B)** as well as the samples of serial histological sections across visual cortical areas **(C)**. A18, A19, A21a, A7, and PMLS represent the visual cortical area 18, 19, 21a, 7, and posterior medial bank of the lateral suprasylvian sulcus, respectively. The dashed lines are estimates of landmarks between different visual areas. The dashed-line circles in **(A)** and **(B)** along the anterior (A)-to-posterior (P) direction in A17 represent locations of electrode penetration. The number on the left of each section in **(C)** indicates the serial section number counted along the posterior-to-anterior direction, and arrow heads indicate sections with visible electrode tracks. The scale bar equals to 2 mm.
