# Supplementary figures and images for: Suppression of top-down influence decreases both behavioral and V1 neuronal response sensitivity to stimulus orientations in cats
Source: Front Behav Neurosci. 2023 Feb 8;17:1061980. doi: 10.3389/fnbeh.2023.1061980 (PMC9944033; doi:10.3389/fnbeh.2023.1061980)

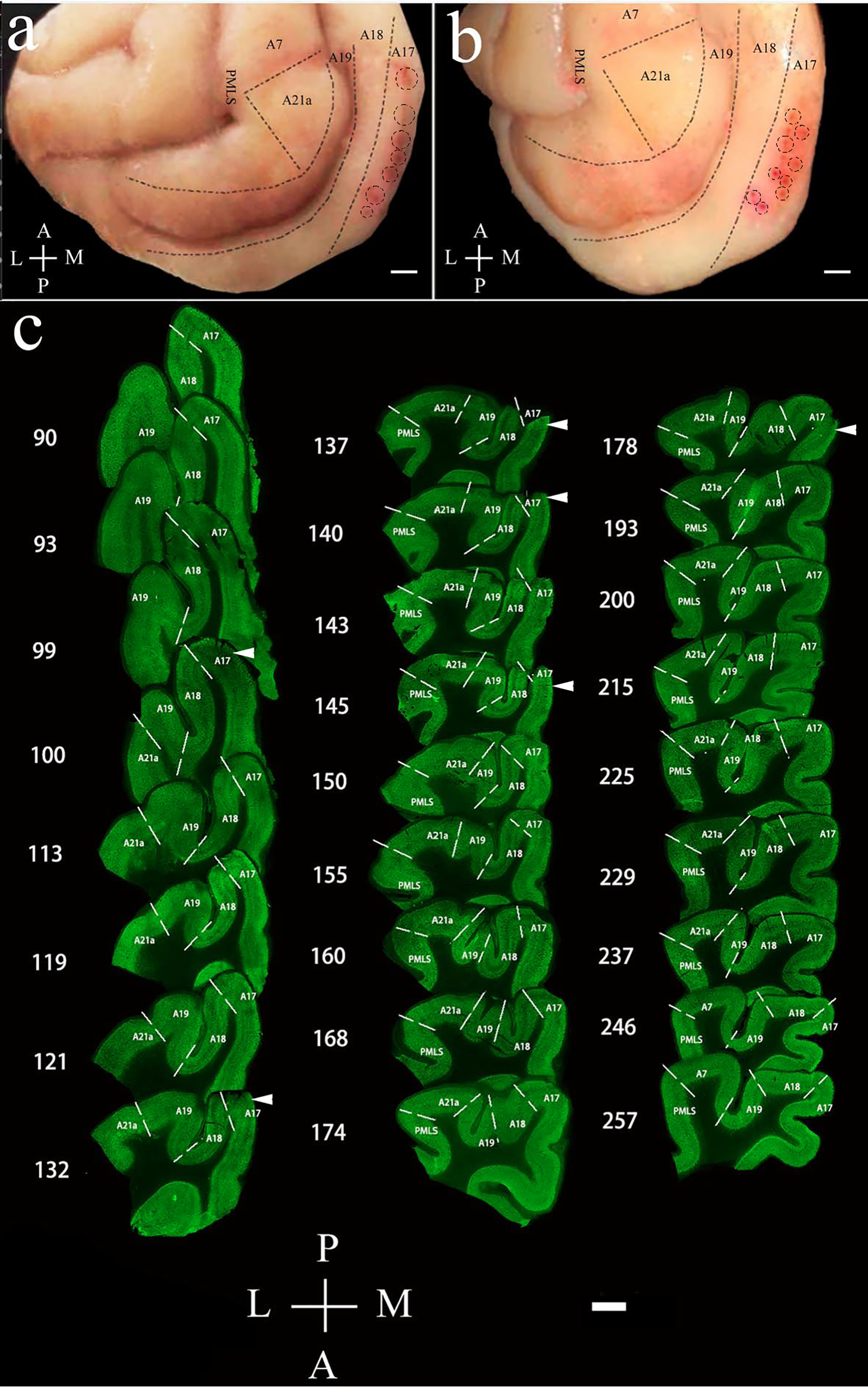

Supplement: Supplementary Figure 1 — Show the recording sites in the V1 cortex (area 17, A17) of cat1 (A) and cat2 (B) as well as the samples of serial histological sections across visual cortical areas (C). A18, A19, A21a, A7, and PMLS represent the visual cortical area 18, 19, 21a, 7, and posterior medial bank of the lateral suprasylvian sulcus, respectively. The dashed lines are estimates of landmarks between different visual areas. The dashed-line circles in (A) and (B) along the anterior (A)-to-posterior (P) direction in A17 represent locations of electrode penetration. The number on the left of each section in (C) indicates the serial section number counted along the posterior-to-anterior direction, and arrow heads indicate sections with visible electrode tracks. The scale bar equals to 2 mm. [file Image_1.TIF]
